# Integration of Tumor Mutation Burden and PD-L1 Testing in Routine Laboratory Diagnostics in Non-Small Cell Lung Cancer

**DOI:** 10.3390/cancers12061685

**Published:** 2020-06-24

**Authors:** Stefanie Schatz, Markus Falk, Balázs Jóri, Hayat O. Ramdani, Stefanie Schmidt, Eva-Maria Willing, Roopika Menon, Harry J. M. Groen, Linda Diehl, Matthias Kröger, Claas Wesseler, Frank Griesinger, Petra Hoffknecht, Markus Tiemann, Lukas C. Heukamp

**Affiliations:** 1 Institut für Hämatopathologie Hamburg, Fangdieckstraße 75A, 22547 Hamburg, Germany; schatz@hp-hamburg.de (S.S.); falk@hp-hamburg.de (M.F.); schmidt@hp-hamburg.de (S.S.); mtiemann@hp-hamburg.de (M.T.); 2 Lung Cancer Network NOWEL, 26129 Oldenburg, Germany; Hayat.Ramdani@Pius-Hospital.de (H.O.R.); c.wesseler@asklepios.com (C.W.); Frank.Griesinger@Pius-Hospital.de (F.G.); Petra.Hoffknecht@niels-stensen-kliniken.de (P.H.); 3 NEO New Oncology GmbH, Gottfried-Hagen-Straße 20, 51105 Cologne, Germany; Jori@newoncology.de (B.J.); Willing@hp-Hamburg.de (E.-M.W.); Menon@newoncology.de (R.M.); 4 Department of Hematology and Oncology, Pius-Hospital Oldenburg, Georgstraße 12, 26121 Oldenburg, Germany; 5 Institute of Experimental Immunology and Hepatology, University Medical Center Hamburg Eppendorf, Martinistraße 52, 20246 Hamburg, Germany; li.diehl@uke.de; 6 Department of Pulmonary Diseases, University of Groningen and University Medical Center Groningen, Hanzeplein 1, 9713 GZ Groningen, The Netherlands; h.j.m.groen@umcg.nl; 7 Onkologische Schwerpunktpraxis, Kröger Ambulante Onkologie, Wiener Straße 1, 27568 Bremerhaven, Germany; praxiskroeger@googlemail.com; 8 Department of Internal Medicine and Pulmonology, Asklepios Klinikum Harburg, Eißendorfer Pferdeweg 52, 21075 Hamburg, Germany; 9 Department of Internal Medicine-Oncology, University of Oldenburg, Georgstraße 12, 26121 Oldenburg, Germany; 10 Germany Department of Thorax Oncology, Niels-Stensen-Kliniken, Franziskus-Hospital Harderberg Alte Rothenfelder Straße 23, 49124 Georgsmarienhütte, Germany

**Keywords:** immuno-oncology, tumor mutational burden, lung cancer, routine diagnostics, driver mutation, PD-L1

## Abstract

In recent years, Non-small cell lung cancer (NSCLC) has evolved into a prime example for precision oncology with multiple FDA-approved “precision” drugs. For the majority of NSCLC lacking targetable genetic alterations, immune checkpoint inhibition (ICI) has become standard of care in first-line treatment or beyond. PD-L1 tumor expression represents the only approved predictive biomarker for PD-L1/PD-1 checkpoint inhibition by therapeutic antibodies. Since PD-L1-negative or low-expressing tumors may also respond to ICI, additional factors are likely to contribute in addition to PD-L1 expression. Tumor mutation burden (TMB) has emerged as a potential candidate; however, it is the most complex biomarker so far and might represent a challenge for routine diagnostics. We therefore established a hybrid capture (HC) next-generation sequencing (NGS) assay that covers all oncogenic driver alterations as well as TMB and validated TMB values by correlation with the assay (F1CDx) used for the CheckMate 227 study. Results of the first consecutive 417 patients analyzed in a routine clinical setting are presented. Data show that fast reliable comprehensive diagnostics including TMB and targetable alterations are obtained with a short turn-around time. Thus, even complex biomarkers can easily be implemented in routine practice to optimize treatment decisions for advanced NSCLC.

## 1. Introduction

In advanced non-small cell lung cancer, immune checkpoint inhibitors (ICIs) targeting the PD-1/PD-L1 axis, have sustainably changed the therapeutic approach of driver mutation negative tumors. ICIs have become available for first- and second-line treatment and represents a next step in the effort to reduce the group of patients receiving systemic chemotherapy. Despite these advances, a limited group of about 20% of NSCLC patients still benefit from ICI. Biomarkers are needed to predict the response outcome before initiation of therapy. As of now, PD-L1 expression has been established as the only predictive biomarker for treatment with ICIs; nevertheless, several factors are limiting its predictive value. Firstly, tumor heterogeneity in terms of PD-L1 tumor expression can be significant and can also change in response to therapy. Secondly, PD-L1-negative tumors have been reported to respond to ICI as well, rendering PD-L1 an imperfect biomarker and urging the need for other biomarkers, such as microsatellite instability (MSI) and TMB [1,2,3,4,5,6,7]. Potentially, TMB may be an independent factor from PD-L1 expression in predicting outcome. TMB is referred to as the total number of somatic mutations that occur in an exome of a tumor genome, although the exact calculation and definition of TMB might differ based on the type of variants, region size, or localization [8,9]. Exonic mutations can lead to the translation of novel peptide epitopes on the tumor surface that may increase the immunogenicity and, therefore, trigger an immune response [9,10,11,12,13]. TMB has been demonstrated to be a strong predictive value for the efficacy of ICI in second- and third-line monotherapy, in first-line monotherapy, and in the first-line combination of immuno-oncological (IO) substances, even if the patients’ tumors did not show expression of PD-L1 [14,15,16,17]. Furthermore, TMB is evaluated for the selection of patients who benefit from the combination of nivolumab and ipilimumab over platinum-based chemotherapy [18]. A recent study described TMB as a predictor for survival after immunotherapy across multiple cancer types, including head and neck, bladder, breast, and renal cancer, underlining the general validity of TMB stratification [19]. Two other recent studies revealed that the efficacy of pembrolizumab plus chemotherapy or placebo plus chemotherapy as first-line treatment was not associated with TMB [20,21]. The lack of correlation between PD-L1-expression and TMB status has been observed in several different studies, highlighting the independent and potential complementary role for both biomarkers [14,15,16,22]. However, the measurement and calculation of TMB still misses uniform standards. Several factors can influence TMB, including the DNA repair capacity and mutation rate. Because current neoantigen-predictive algorithms are imperfect, it is likely that the relationship between TMB and antigenicity is complex. Furthermore, additional factors can affect immunogenicity, including the clonality of neoantigens and the tumor microenvironment [9]. In addition, tumor heterogeneity and clonal architecture, the size of the selected genomic region of interest, and setup regarding driver mutational panel bias or even tumor purity can also influence the results from a biological perspective. From the technical point of view, NGS-derived deamination artifacts, the lack of predefined cut-off values; and the mutation types challenge the introduction of TMB as a general biomarker for immunotherapies [23]. The clinical utility of TMB is also affected by pre- and postanalytic parameters, such as storage of the formalin fixed paraffin embedded (FFPE) sample, turn-around time (TAT), or analysis failure rate [24,25]. To evaluate the importance of TMB as a practical biomarker, clinical routine data are needed. Here, we present the first TMB mono-centric dataset from a cohort of 417 lung cancer samples in Germany. We aimed to cover the proposed gaps by discussing clinical usability as driver mutations and TMB in the period from 2018 to 2020 using a commercially available assay (NEOplus v2 RUO^‡^, NEO New Oncology GmbH). The assay was designed to detect targetable driver mutations and genomic alterations (i.e., translocations, copy number changes) of other clinically relevant genes, such *KRAS*, *KEAP1*, *STK11*, and *ARIDA1* (Appendix A), as well as to estimate TMB in an exonic territory of 1.14 Mb. After internal validation, the assay was used in our accredited clinical laboratory for routine mutation analysis in NSCLC patients. Samples were exclusively tested upon request by the attending physician and therefore reflect real world data 

## 2. Results

### 2.1. TMB Assay Correlation with Clinical Trail Assay of CheckMate 227

To determine whether the assay was able to estimate TMB comparable to the assay used in the CheckMate 227 clinical trial, a series of 17 samples were analyzed both in-house as well as by F1CDx (Foundation Medicine Inc., Cambridge Massachusetts) 

**Figure 1:**
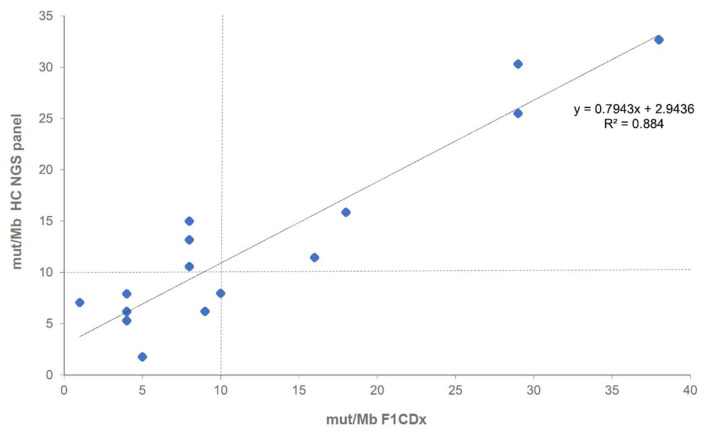
Correlation of TMB estimation of 17 samples measured by NEOplus v2 RUO^‡^ and F1CDx assays.

A high correlation (R^2^ = 0.884, 95% CI [0.799, 0.968]) between both assays was observed and the TMB category (high vs. low) showed 80% (12/15) concordance with the F1CDx category with a cut-off of 10 mut/Mb (Figure 1). 

### 2.2. Histology, Driver Mutations, and PD-L1

In this cohort, 42.4% (177/417) of patients were of female gender and 57.6% (240/417) were male, the mean age being 66 years (Table 1). Histological classification revealed 73.9% (308/417) adeno carcinomas, 0.2% (1/417) adeno-squamous, 7.9% (33/417) squamous cell, 1.0% (4/417) small cell lung cancer, 0.5% (2/417) of cases displayed large cell neuroendocrine differentiation, while 16.5% (69/417) were not otherwise specified (NOS). *EGFR* mutations were detected with a frequency of 14.87% (62/417); however, only 66% (41/62) constituted the classical targetable drivers including exon 19 deletions or L858R. The remaining 34% were mostly located outside exons 18–21. Exon 20 insertions, resistant to first-, second-, and third-generation EGFR tyrosine kinase inhibitors (TKIs), were detectable in six cases (6/417, 1.4%) (Table 1). 

Several point mutations were detected within the *BRAF* gene (36/417, 8.63%); among these, nine were *BRAF* V600E. Deemed targetable gene fusions were found with a frequency of 9.83% (41/417), including 15 (3.66%) *EML4-ALK*, 2 *CD74-ROS1* (0.48%), and 3 *RET* translocations (0.73%, 2x *KIF5B-RET*, *RET-CCDC6*). Several other fusions were detected, including inter- and intragenic fusions of *TP53*, *RB1*, *STK11*, and *CDKN2A/B*. Within the group of adenocarcinomas, 79.9% of cases were devoid of a targetable driver alteration and 1.6% showed wildtype in all 39 therapeutically relevant genes. None of the squamous cell carcinomas showed a targetable driver alteration. In parallel to mutational analysis, PD-L1 immunohistochemistry (IHC) was performed and the tumor proportion score (TPS) was determined for each sample. In total, 123/417 (29.5%) of tumors did not express PD-L1 (<1% of PD-L1), while 11.99% showed PD-L1 expression between ≥1% and <5% PD-L1, 18.94% between ≥5% and <50% PD-L1, and 23.74% of cases had strong PD-L1 expression (≥50% PD-L1). In 15.8% of cases, no PD-L1 analysis was performed either because it was not requested by the physician or due to limited tissue availability.

### 2.3. Turn-Around Time and HC NGS Workflow

**Figure 2:**
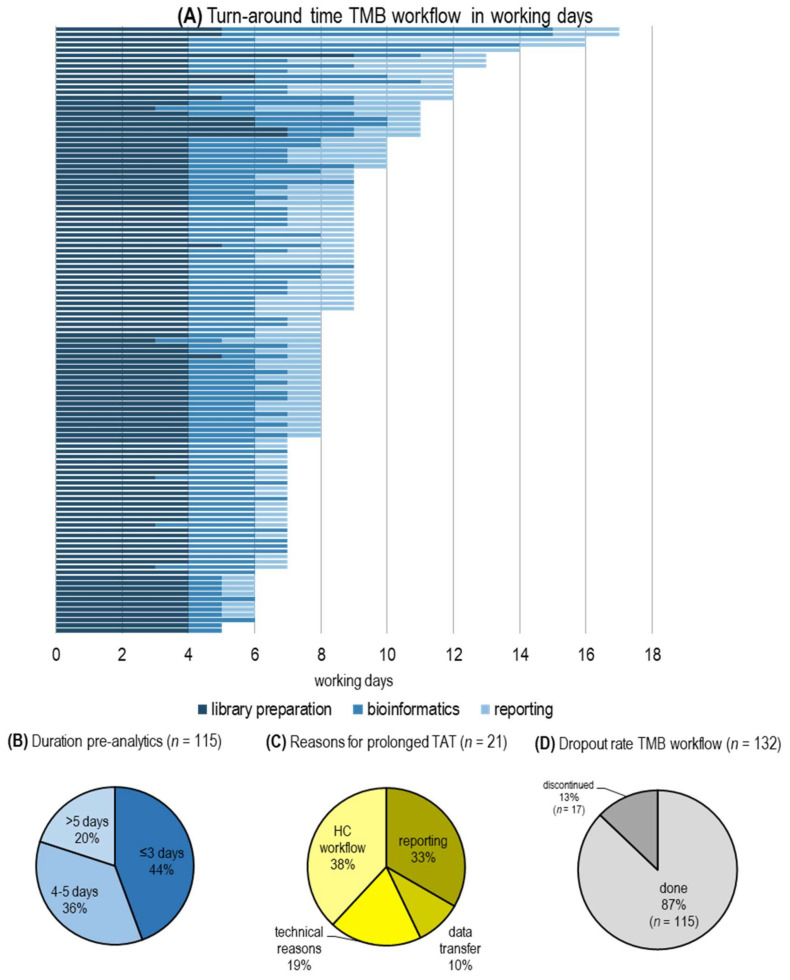
Turn-around time for TMB evaluation. (**A**) TAT in working days per case from start of HC NGS workflow to reporting; (**B**) Duration of pre-analytics including tissue embedding and DNA extraction; (**C**) Reasons for prolonged TAT (>10 working days); (**D**) Percentage of cases not meeting quality criteria.

According to European guidelines, turn-around times for molecular testing in advanced lung cancer should take no longer than 10 working days [27,28]. We initially wanted to determine whether comprehensive testing including TMB estimation was feasible within this timeframe. To that end, we analyzed the first 115 cases for turn-around time and found that from 81.7% (94/115) of cases a result was sent to the treating physician within 10 working days (Figure 2). To further improve on the turn-around time, we looked at the steps responsible for possible delays more closely. The HC NGS workflow was subdivided into library preparation, sequencing, bioinformatics (data processing), and generation of a pathological report. Underlying causes for extended turn-around times in these 21 cases were delays in reporting (33%) mainly due to complex genomic alterations; library preparation (38%), such as insufficient DNA at intermediate steps of library preparation requiring some step to be repeated; other technical issues (19%), such as operator or handling mistakes; or delays in data handling and bioinformatics due to server drop out (10%) (Figure 2). Pre-analytics, including FFPE embedding, histological evaluation for tumor content and DNA extraction, required between 2 to 5 days in 80% of cases. More than 5 days were needed in 20% of samples due to delays in workflow (Figure 2). As only one HC NGS run was performed initially per week, the sample entry date also influenced overall TAT.

### 2.4. Evaluation of Tumor Mutation Analysis in Routine Lab Samples

#### 2.4.1. TMB in Relation to Age

**Figure 3:**
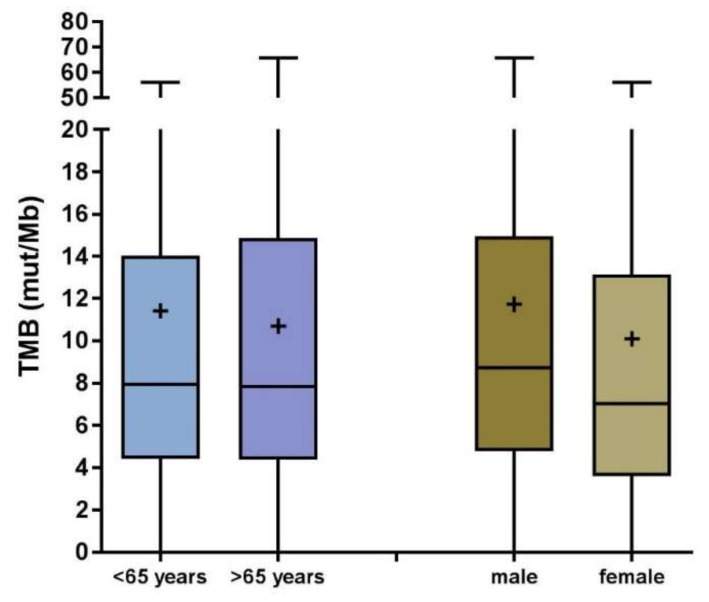
TMB status (mut/Mb) is not significantly correlated to age or gender. Boxplots are shown with the 95% confidence interval indicated by the box. Lines indicate the mean and + the median. Statistical analysis by Student *t* test did not reveal significant differences in TMB between patients under or over 65 years of age (*p* = 0.476), nor between male and female patients (*p* = 0.110).

Based on these findings, we considered the assay set up sufficiently fast to continue using the assay for further routine diagnostics including targetable mutations. After a total of 417 were analyzed, we correlated patient-specific parameters with the observed TMB values. There appeared no significant age- (*p*= 0.476) or gender (*p* = 0.110)-specific association with TMB (Figure 3).

#### 2.4.2. TMB in Relation to Driver Mutations

Recent reports suggest that the presence of typical driver alterations inversely correlates with the number of somatic tumor mutations [29,30]. In our cohort, targetable driver alterations (*EGFR*, *ALK*, *ROS*, *BRAF* V600E, *RET*) occurred at a combined frequency of 17.5% (73/417). The majority of this group of patients 62/73 (84.9%) showed a lower TMB, below a cut-off of 10 mut/Mb, while only 11/73 (15.1%) patients were in the TMB-high group (≥10mut/Mb).

**Figure 4:**
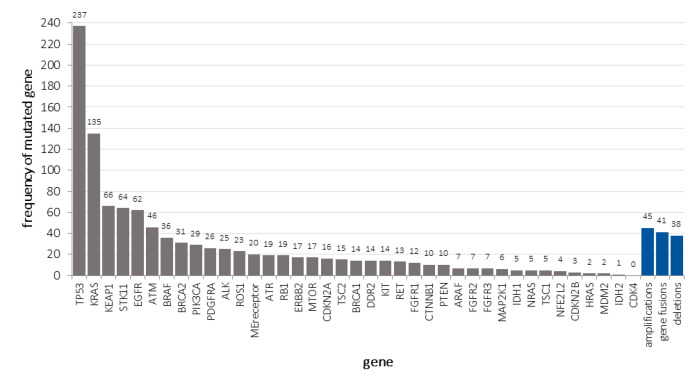
Number of tumors carrying non-synonymous gene mutations in descending order of frequency (grey), including gene amplifications, fusions, and deletions (blue).

The most prevalent somatic mutations found were *TP53* (237/417, 56.8%), followed by *KRAS* (135/417, 32.4%), *KEAP1* (54/417, 15.8%), *STK11* (64/417, 15.3%), *EGFR* (62/417, 14.9%), *ATM* (46/417, 11.03%), and others. Gene amplifications were discovered in 45/417 (10.8%) and gene fusions in 9.83% (41/417) of cases (Figure 4).

#### 2.4.3. TMB and PD-L1

**Figure 5:**
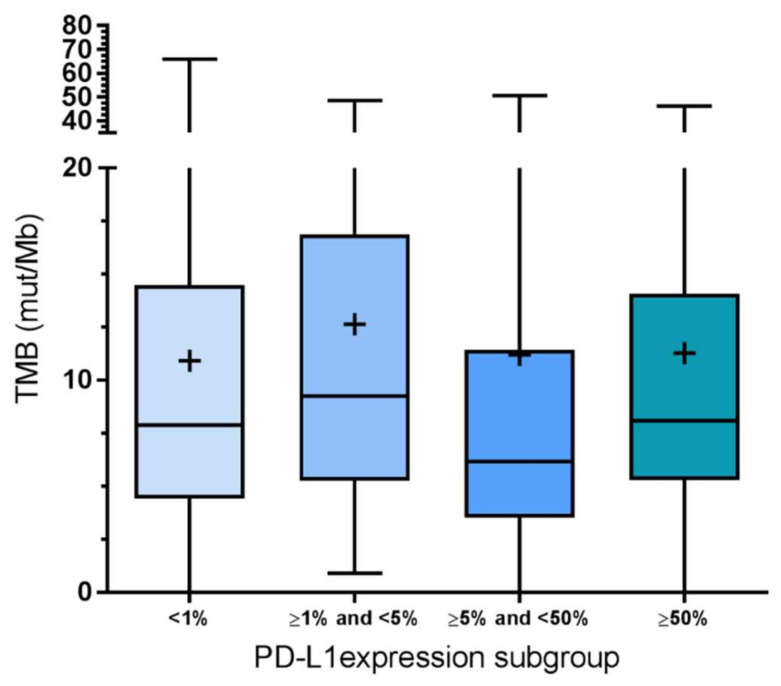
TMB is not different in the four groups of the PD-L1 tumor proportion score. Data are presented as box plots with a 95% confident interval. The line indicates the mean, the + indicates the median. Statistical analysis by ANOVA did not reveal significant (*p* < 0.05) differences in TMB between TPS groups.

Data from the literature indicate that PD-L1 expression on tumor cells does not correlate with TMB and that TMB should therefore be considered as an independent predictive biomarker [31]. In line with this observation, we found no significant association between the TMB value and PD-L1 TPS (Figure 5). Further, we categorized the tumors on the basis of PD-L1 tumor proportion scores and observed no statistically significant association (as assessed by analysis of variance, ANOVA) of PD-L1 tumor expression and TPS scores (*p* = 0.798).

#### 2.4.4. Correlation of Somatic Tumor Mutations and TMB

**Figure 6:**
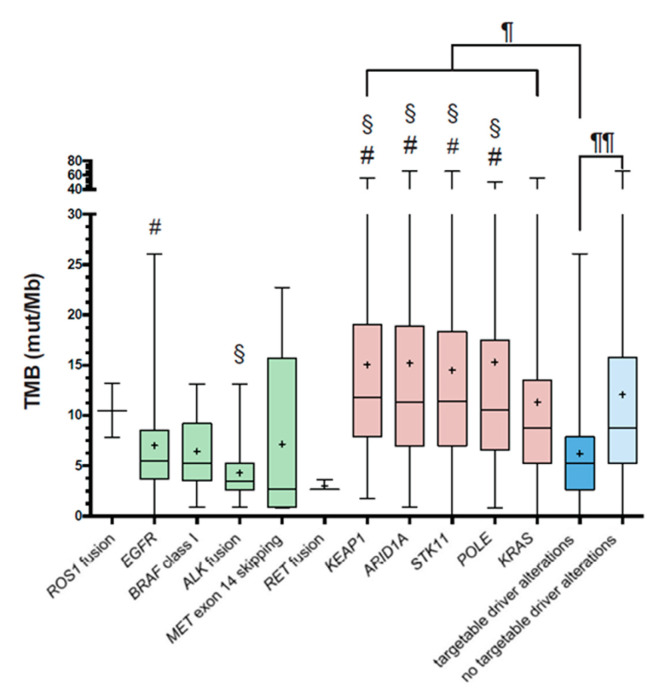
TMB and clinically relevant genetic alterations. For targetable driver alterations (green and blue), TKI-sensitive mutations were counted. Regarding *KEAP1*, *ARID1A*, *STK11*, and *POLE*, all non-synonymous aberrations (red) were considered. Data are presented as box plots with a 95% confident interval. The line indicates the mean, the + indicates the median. Statistical analysis by ANOVA revealed significant differences in TMB between *EGFR* and *KEAP1*, *ARID1A*, *POLE*, and *STK11* (#) mutated groups; between *ALK* fusion and *KEAP1*, *ARID1A*, *POLE*, and *STK11* mutated groups (§); and between targetable driver mutations and all red groups (¶); and lastly, between targetable and non-targetable driver mutation groups (¶¶).

It has been shown that the presence of targetable driving mutations, such as activating *EGFR* mutations, is associated only with limited responses to immune checkpoint inhibitors [32]. It has been postulated that this is due to low TMB. Particularly, *EGFR* and *ALK* mutations are more frequent in light or never smokers, further supporting this hypothesis [33]. Tumors characterized by a targetable driver alteration show significantly lower TMB compared to those lacking any driver alteration (*p* < 0.001). TMB in *EGFR* mutated tumors was significantly lower compared to TMB in *KEAP1* (*p* < 0.01), *ARID1A* (*p* < 0.05), *STK11* (*p* < 0.05), and *POLE* (*p* < 0.05) mutated tumors. Along those lines, TMB in *ALK* mutated tumors was significantly lower compared to *KEAP1*, *ARID1A*, *POLE*, and *STK11* mutated tumors (each *p* <0.05) (see Figure 6).

### 2.5. Clinical Use of IO-Related Biomarkers in Clinical Decision-Making

Recent publications suggest that *STK11* and *KEAP1* mutations might be associated with an inferior response to IO [34,35]. Contrarily, *ARID1A* mutations seem to confer positive prediction [36]. Based on these findings, we observe that clinicians are interested in additional biomarkers with predictive value for IO. 

Inactivating mutations in *STK11* were detected with a frequency of 11.3% (47/417) and for *KEAP1* with 7.7% (32/417). Functional mutations in *ARID1A* were found in 5.3% (22/417) of patients. Interestingly, all these variants were associated with higher TMB values, indicating that particularly *STK11* and *KEAP1* are biomarkers independent from TMB.

## 3. Discussion

When TMB emerged as a potential predictive biomarker, it was considered to possibly be the most challenging and complex genomic biomarker to date. NGS-based sequencing of a minimum of one Megabase exonic territory in combination with advanced bioinformatics analyses is needed. The NEOplus v2 RUO^‡^ panel has a total territory of 2.50 Mb, of which 1.14 Mb are dedicated to TMB evaluation. The territory dedicated to TMB evaluation excludes typical cancer genes that are known to have a mutation bias. The need for a TMB territory size of greater than 1 Mb is based on the data of Chalmers et al. and generally considered to be sufficient. This is supported by the comparisons of several TMB assays in the recently published German comparative TMB study [37]. There was considerable doubt whether speedy local TMB testing could be implemented and whether testing large number of patients was feasible. The initiatives of Quality in Pathology and Friends of Cancer Research jointly addressed the need for harmonization of TMB testing. In this study on a cohort of 417 lung cancer patients, we asked whether TMB can be assessed alongside other predictive genomic biomarkers needed for treatment stratification. We implemented an HC NGS assay into routine diagnostics, allowing for simultaneous detection of TMB and relevant aberrations including targetable driver mutations. TMB values correlated well with the F1Dx panel. These data are compatible with previously published data from a German harmonization trial [24,37].

The TAT proved to be in line with guidelines as 81.7% of cases met the required turn-around time of 10 working days. Comparing the NEOplus v2 RUO^‡^ HC assay to the F1Dx assay (used in CheckMate 227) revealed a high degree of association.

Samples analyzed in this study mainly consisted of adenocarcinomas; however, a significant proportion of biopsies were histologically classified in external pathologies and the histological subtype was not reported to us. Therefore, a relatively large proportion appeared as NOS; unfortunately, in most cases, tissue was not sufficient for reevaluation. TMB was not associated to age in our cohort, although previously, an age effect was published regarding other cancer entities [38]. TMB and PD-L1 expression was not associated, supporting the concept of two independent and therefore potentially additive biomarkers for immune oncological treatment. Male gender appears to be associated with higher TMB compared to females, potentially reflecting different smoking habits; however, this difference was not statistically significant. The frequency of targetable driver alterations (*EGFR*, *ALK*, *ROS1*, *MET* exon 14 skipping, *RET*, or BRAF V600E) was in line with the literature and their presence was significantly associated with a lower TMB value. In contrast, tumors that carried mutations in KRAS or genes assumed to cause primary resistance to IO (*STK11*, *KEAP1*, and *POLE*) showed higher TMB. In 2019, the complex CheckMate 227 trial using TMB as a co-primary endpoint in some subgroups could not convincingly show a role for TMB as a useful predictive biomarker. Since then retrospective analyses explored the use of pembrolizumab versus chemotherapy in all therapeutic lines, showing improvement in OS, PFS, and ORR for TMB-high patients [39,40].

Based on the KEYNOTE-158 trial, the FDA recently approved pembrolizumab monotherapy for patients with solid tumors and TMB ≥10 mut/Mb. The study included adults and pediatric patients with unresectable or metastatic disease with progression upon prior treatment or no alternative treatment options. This underlines the concept of TMB as a predictive biomarker [41].

## 4. Materials and Methods 

### 4.1. Selection of Patients

Patients were retrospectively selected from our internal pathological documentation system based on the request for comprehensive molecular testing from 2018 to 2019. All samples represent tumor biopsies tested in a single institution (Institut für Hämatopathologie Hamburg, Germany). Use of anonymized patient data was reviewed by the local ethics committee (Ref number: WF-055/18 and WF-017/19.)

### 4.2. PD-L1

PD-L1 was stained immunohistochemically using the antibody clone 22C3 pharm Dx (Dako Omnis, 1:30 dilution) on the automated BenchMark Ultra platform (Roche Diagnostics) with positive controls of the spleen, tonsil, and placenta as part of a multi-tissue control. Scoring was conducted by board-certified and trained pathologists [42].

### 4.3. Mutation Testing

For molecular analysis, 3–10 5–10µm micrometer formalin-fixed paraffin-embedded (FFPE) sections were prepared and tumor tissue was micro-dissected when the tumor content was below 10%. DNA was extracted semi-automated (Maxwell® 16, Promega), and 400ng of input DNA was sonographically sheared (Covaris®) into approximately 200-bp double-stranded fragments. Hereafter, adapters were ligated, and genomic regions of interest were enriched using complementary bait sequences. During this hybrid capture, the selected baits ensure optimal coverage of all relevant genomic regions, including 340 genes in a 1.14 Mb complete genomic territory size. Following the enrichment, the targeted fragments were clonally amplified and sequenced with next-generation sequencing (NextSeq 500/550, Illumina). Point mutations, small insertions and deletions, copy number alterations, and rearrangement/gene fusions were identified with NEO New Oncology’s proprietary computational biology analysis pipeline and analyzed using the NEO diagnosis software. 

### 4.4. TMB

For TMB, the number of somatic mutations detected within 1.14 Mb of the genome were quantified and that value extrapolated to the whole exome using a validated algorithm (NEO New Oncology). Alterations known to be included in genomic databases, such as Single Nucleotide Polymorphism Database (dbSNP) or Exome Aggregation Consortium (ExAc), were excluded. 

TMB was calculated from genomic alterations identified by the bioinformatics pipeline. Single nucleotide changes with predicted missense, nonsense, silent, nonstop consequences, and small insertions/deletions with in-frame or frameshift insertion/deletion consequences were considered for TMB calculation. Variants with an allelic frequency of at least 5% (for LOD 0.05 value) or 10% (for LOD 0.1 value) were included and frequent germ line variants present in ExAc and dbSNP were not considered. The TMB value was provided as mutations per Megabase (mut/Mb).

### 4.5. Statistical Analysis

Descriptive statistics were used. Differences between groups were tested with parametric or non-parametric methods depending on the distribution. Box plots were generated using GraphPad Prism 7.04 (GraphPad Software, Inc., San Diego, CA) and statistical significance assessed by Student *t* and ANOVA (Alpha level 0.05). For Figure 1, Pearson correlation was performed using Microsoft Excel.

## 5. Conclusions

We showed that routine diagnostics of highly complex biomarkers, such as TMB, is feasible today and that a central industrial testing facility is not required. This is a highly important finding given that clinical trial results using TMB as a predictive biomarker are promising and FDA approval is currently pending. However, at this point in time, data are still not fully mature and further validation is required. 

## Figures and Tables

**Table 1:** Overview of patients’ characteristics according to TMB values.

Variation		Total		TMB < 10		TMB > 10	
		*N* = 417		*N* = 260	(62.35%)	*N* = 157	(37.65%)
Age	Median	66		66		66	
	*Mean (±SD)*	*65.0*	(11.8)	64.7	(12.5)	65.7	(10.5)
	*Range*	*21–92*		*21–92*		*93–90*	
	<65 years	199	(47.72%)	126	(48.46%)	73	(46.50%)
	≥65 years	218	(52.28%)	134	(51.54%)	84	(53.50%)
Sex	Female	177	(42.45%)	140	(53.85%)	100	(63.69%)
	Male	240	(57.55%)	120	(46.15%)	57	(36.31%)
Histology	Adenocarcinoma	308	(73.86%)	201	(77.31%)	107	(68.15%)
	Squamous	33	(7.91%)	14	(5.38%)	19	(12.10%)
	Adeno-squamous	1	(0.24%)	1	(0.38%)		
	Large-cell neuroendocrine	2	(0.48%)			2	(1.27%)
	SCLC	4	(0.96%)	4	(1.54%)		
	NOS	69	(16.55%)	40	(15.38%)	29	(18.47%)
*EGFR* Status	Mutant	62	(14.87%)	47	(18.08%)	15	(9.55%)
	Wild type	355	(85.13%)	213	(81.92%)	142	(90.45%)
	targetable *EGFR* mutation	41	(66.13%)	33	(12.69%)	8	(5.10%)
	targetable *EGFR* plus resistance mutation T790M	3	(4.84%)	3	(1.15%)		
	*EGFR* exon 20 insertion	6	(9.68%)	6	(2.31%)		
	other / variant of unknown significance	12	(19.35%)	5	(1.92%)	7	(4.46%)
*BRAF* Status	Mutant	36	(8.63%)	20	(7.69%)	16	(10.19%)
	Wild type	381	(91.37%)	240	(92.31%)	141	(89.81%)
	V600E / class I *	9	(25.00%)	8	(3.08%)	1	(0.64%)
	non-V600E / class II *	11	(30.56%)	5	(1.92%)	6	(3.82%)
	non-V600E / class III *	5	(13.89%)	3	(1.15%)	2	(1.27%)
	other mutation / variant of unknown significance	11	(30.56%)	4	(1.54%)	7	(4.46%)
Gene Fusions	Mutant	41	(9.83%)	32	(12.31%)	9	(5.73%)
	Wild type	368	(88.25%)	223	(85.77%)	145	(92.36%)
	n.d.	8	(1.92%)	5	(1.92%)	3	(1.91%)
	*ALK* translocation	15	(36.59%)	14	(5.38%)	1	(0.64%)
	*ROS1* translocation	2	(4.88%)	1	(0.38%)	1	(0.64%)
	*RET* translocation	3	(7.32%)	3	(1.15%)		
	other fusions / translocation of unknown significance	21	(51.22%)	14	(5.38%)	7	(4.46%)
PD-L1 TPS	<1%	123	(29.50%)	79	(30.38%)	44	(28.03%)
	≥1% and <5%	50	(11.99%)	26	(10.00%)	24	(15.29%)
	≥5% and <50%	79	(18.94%)	55	(21.15%)	24	(15.29%)
	≥50%	99	(23.74%)	53	(20.38%)	46	(29.30%)
	n.d.	66	(15.83%)	47	(18.08%)	19	(12.10%)

* *BRAF* mutations were classified based on Yao et al. [26].

## Appendix A

List of genes included in NEOplus v2 RUO^‡^. NEOplus v2 RUO^‡^ detects point mutations in 330 genes and small insertions and deletions in 325 genes, as well as copy number changes in 230 genes. In addition, gene fusions are detected in 16 genes.

^‡^ For Research Use Only. Not for use in diagnostic procedures.

Point Mutations, small Insertions and Deletions

ABL1, ABL2, ACVR1B, AKT1, AKT2, AKT3, ALK, ALMS1, AMER1, APC, APLNR, AR, ARAF, ARHGEF12, ARID1A, ARID1B, ARID2, ASXL1, ATAD5*, ATM, ATR, ATRX, AURKA, AURKB, AXIN2, AXL, B2M, BAP1, BARD1, BCL2, BCL2L1, BCL6, BLM, BMPR1A, BMS1, BRAF, BRCA1, BRCA2, BRD2, BRD3, BRD4, BRIP1, BTK, BUB1B, CARD11, CASP8, CBFB, CBL, CCND1, CCND2, CCNE1, CD274, CD58, CD79A, CD79B, CDC73, CDH1, CDK12, CDK4, CDK6, CDK8, CDKN1A, CDKN1B, CDKN2A, CDKN2B, CDKN2C, CEBPA, CHD4, CHEK1, CHEK2, CIC, CLSPN, CREBBP, CRKL, CSF1R, CTCF, CTNNA1, CTNNB1, CUL3, DAXX, DCUN1D4, DDB2, DDR2, DICER1, DOT1L, EGFR, EMSY, EP300, EPHA3, EPHA5, EPHA7, EPHB1, ERBB2, ERBB3, ERBB4, ERCC3, ERCC4, ERCC5, ERCC6, ERRFI1, ESR1, EZH2, FANCA, FANCB, FANCC, FANCD2, FANCE, FANCF, FANCG, FANCI, FANCL, FANCM, FAS, FAT1, FBXW7, FGFR1, FGFR2, FGFR3, FGFR4, FLCN, FLT1, FLT3, FOXO3, FOXP1, FRS2, GATA1, GEN1, GLI1, GNA11, GNA13, GNAI2, GNAQ, GNAS, GNAT2, GSK3B, H3F3A, H3F3B, HDAC2, HGF, HLTF, HRAS, HSP90AA1, IDH1, IDH2, IFNGR1, IFNGR2, IGF1R, IGF2, IGF2R, IKBKB, IKBKE, IL21R, INHBA, INPP4B, IRF1, IRF2, IRF4, JAK1, JAK2, JAK3, JUN, KAT6A*, KDR, KEAP1, KIT, KMT2A, KMT2B, KMT2C, KMT2D, KRAS, KSR1, LRP1B, LYN, MAD2L1, MAGI2, MAP2K1, MAP2K3, MAP2K4, MAP3K1, MCL1, MDM2, MDM4, MED12, MEN1, MET, MITF, MLANA, MLH1, MLH3, MPL, MRE11, MSH2, MSH3, MSH5, MSH6, MST1R, MTOR, MUTYH, MYC, MYCL, MYCN, NBN, NCOA3, NCOA4, NF1, NF2, NFE2L2, NFKBIA, NOTCH2, NOTCH4, NPM1, NRAS, NSD1, NTRK1, NTRK2, NTRK3, PALB2, PBRM1, PDCD1LG2, PDGFRA, PDGFRB, PDK1, PIK3C2B, PIK3CA, PIK3CB, PIK3CD, PIK3CG, PIK3R1, PIK3R2, PLCG2, PMS2, POLD1, POLE, POLG, POLH, POT1, PPM1D, PPP2R1A, PRDM1, PREX2, PRKCI, PRKDC, PRKN, PSMB5, PTCH1, PTEN, PTPN11, PTPRC, PTPRK, PTPRT, QKI, RAC1, RAD50, RAD51, RAD51B, RAD51C, RAD51D, RAD54L, RAD54L2, RAF1, RANBP2, RARA, RB1, RECQL4, RET, REV3L, RICTOR, RIT1, RNF43, RNPS1, ROS1, RPL10A, RPL23, RPTOR, SDHA*, SDHAF2, SDHB, SDHC, SDHD, SERPINB3, SERPINB4, SETD1A*, SETD1B, SETD2, SF3B1, SLIT2, SLX4, SMAD2, SMAD3, SMAD4, SMARCA4, SMARCB1, SMO, SOX2, SPEN, SPOP, SRP54, STAG2, STAT3, STK11, SUFU, SYK, TAF3, TAP1, TAP2, TAPBP, TCF7L2, TGFBR2, TNFAIP3, TNFRSF14, TOP1, TOP2A, TP53, TP53BP1, TRRAP, TSC1, TSC2, TSHR, U2AF1, U2AF1L5, VEGFA, VHL, WISP3, WRN, WT1, XPA, XPC, XRCC1, ZFHX3*, ZNF217

* Detection of small insertions and deletions in ATAD5, KAT6A, SDHA, SETD1A, ZFHX3 not possible.

Copy Number Changes

ABL1, AKT1, AKT2, AKT3, ALK, ALMS1, AMER1, APC, APLNR, AR, ARAF, ARID1A, ARID1B, ARID2, ASXL1, ATAD5, ATM, ATR, ATRX, AURKA, AXIN2, AXL, BARD1, BCL6, BLM, BMPR1A, BMS1, BRAF, BRCA1, BRCA2, BRD2, BRD3, BRD4, BRIP1, BTK, BUB1B, CARD11, CASP8, CCND1, CCNE1, CD274, CDC73, CDH1, CDK12, CDK4, CDK6, CDK8, CDKN2A, CDKN2B, CHD4, CIC, CLSPN, CREBBP, CSF1R, CTNNA1, CTNNB1, CUL3, DAXX, DDB2, DDR2, DICER1, DOT1L, EGFR, EMSY, EP300, EPHA3, EPHB1, ERBB2, ERBB3, ERBB4, ERCC3, ERCC4, ERCC5, ERCC6, ERRFI1, ESR1, EZH2, FANCA, FANCB, FANCC, FANCD2, FANCE, FANCI, FANCL, FANCM, FAS, FAT1, FGF19$, FGF23#, FGF3$, FGF4$, FGF6#, FGFR1, FGFR2, FGFR3, FGFR4, FLT1, FLT3, FOXO3, FOXP1, FRS2, GATA1, GLI1, GSK3B, HDAC2, IDH1, IDH2, IFNGR1, IKBKB, IKBKE, IL21R, INHBA, INPP4B, IRF1, IRF2, IRF4, JAK1, JAK2, JAK3, KAT6A, KDR, KEAP1, KIT, KRAS, LRP1B, LYN, MAGI2, MAP2K1, MDM2, MDM4, MED12, MEN1, MET, MITF, MLH1, MPL, MRE11, MSH2, MSH3, MSH5, MSH6, MST1R, MUTYH, MYC, MYCL, MYCN, NBN, NCOA3, NCOA4, NF1, NF2, NFE2L2, NPM1, NSD1, NTRK1, NTRK2, NTRK3, PALB2, PBRM1, PDGFRA, PDGFRB, PDK1, PIK3C2B, PIK3CA, PIK3CB, PIK3CD, PIK3CG, PIK3R1, PIK3R2, PMS2, POLD1, POLE, POLG, POLH, PPM1D, PRDM1, PTCH1, PTEN, PTPN11, PTPRK, QKI, RAD50, RAD51, RAD51B, RAD51C, RAD51D, RAD54L, RAD54L2, RAF1, RANBP2, RARA, RB1, REV3L, RICTOR, RIT1, RNF43, ROS1, RPTOR, SETD1A, SETD1B, SETD2, SF3B1, SLIT2, SLX4, SMAD2, SMAD3, SMAD4, SMARCA4, SMO, SPOP, STAG2, STAT3, STK11, SYK, TAP1, TAP2, TAPBP, TGFBR2, TNFAIP3, TOP1, TOP2A, TP53, TP53BP1, TRRAP, TSC1, TSC2, VEGFA, WRN, XPC, XRCC1

$ genes considered as one cluster

# genes considered as one cluster

Gene Fusions

ALK, BRAF, CD74, EML4, ETV6 (NTRK3 Fusion), FGFR1, FGFR2, FGFR3, KIF5B, MET, NRG1, NTRK1, PDGFRA, RET, ROS1
